# Right Bundle Branch Conduction in Left Bundle Branch Area Pacing

**DOI:** 10.1002/joa3.70331

**Published:** 2026-04-06

**Authors:** Takefumi Fujito, Kento Erata, Naoto Noumura, Atsushi Mochizuki, Masato Furuhashi

**Affiliations:** ^1^ Department of Cardiovascular‐Kidney‐Metabolic Medicine Sapporo Medical University School of Medicine Sapporo Japan; ^2^ Division of Clinical Engineering Sapporo Medical University Hospital Sapporo Japan

**Keywords:** left bundle branch area pacing, right bundle branch block, right bundle branch conduction

## Abstract

The electrical activation during left bundle branch area pacing can penetrate into the right bundle branch, resulting in right ventricular activation. These findings suggest that conventional ECG indicators for confirming left bundle branch capture should be reconsidered when the right bundle branch contributes to right ventricular activation.

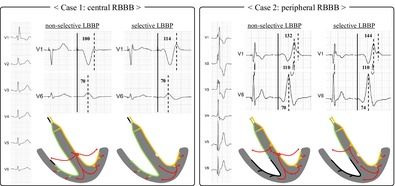

The left ventricle (LV) is activated earlier than the right ventricle (RV) during left bundle branch area pacing (LBBAP), and the terminal R wave is therefore typically present in lead V1 [1]. However, the involvement of right bundle branch (RBB) activation during LBBAP has not been clarified yet.

Case 1 was a 78‐year‐old male patient who underwent pacemaker implantation for a diagnosis of 2:1 atrioventricular block (AVB). A preoperative 12‐lead electrocardiogram showed 2:1 AVB with right bundle branch block (RBBB) (Figure 1A). We implanted a Tendril STS pacing lead (Abbott, Sylmar, CA) using a CPS Locator 3D catheter (Abbott) and attempted LBBAP (Figure 1B). In the endocardial electrogram, no distinct left bundle branch (LBB) potential was observed during intrinsic conduction. During the threshold test with bipolar pacing, the interval from the pacing stimulus to the peak of the R wave in lead V6 (Stim‐V6RWPT) remained unchanged at 70 ms, whereas the interval from the pacing stimulus to the peak of the R wave in lead V1 (Stim‐V1RWPT) was prolonged from 100 to 114 ms (Figure 1C,D). In addition, with the prolongation of Stim‐V1RWPT, a discrete potential was observed after the pacing stimulus in the endocardial electrogram (Figure 1D). Based on these findings, a transition from nonselective left bundle branch pacing (NS‐LBBP) to selective left bundle branch pacing (S‐LBBP) during the threshold test was suggested.

**FIGURE 1:**
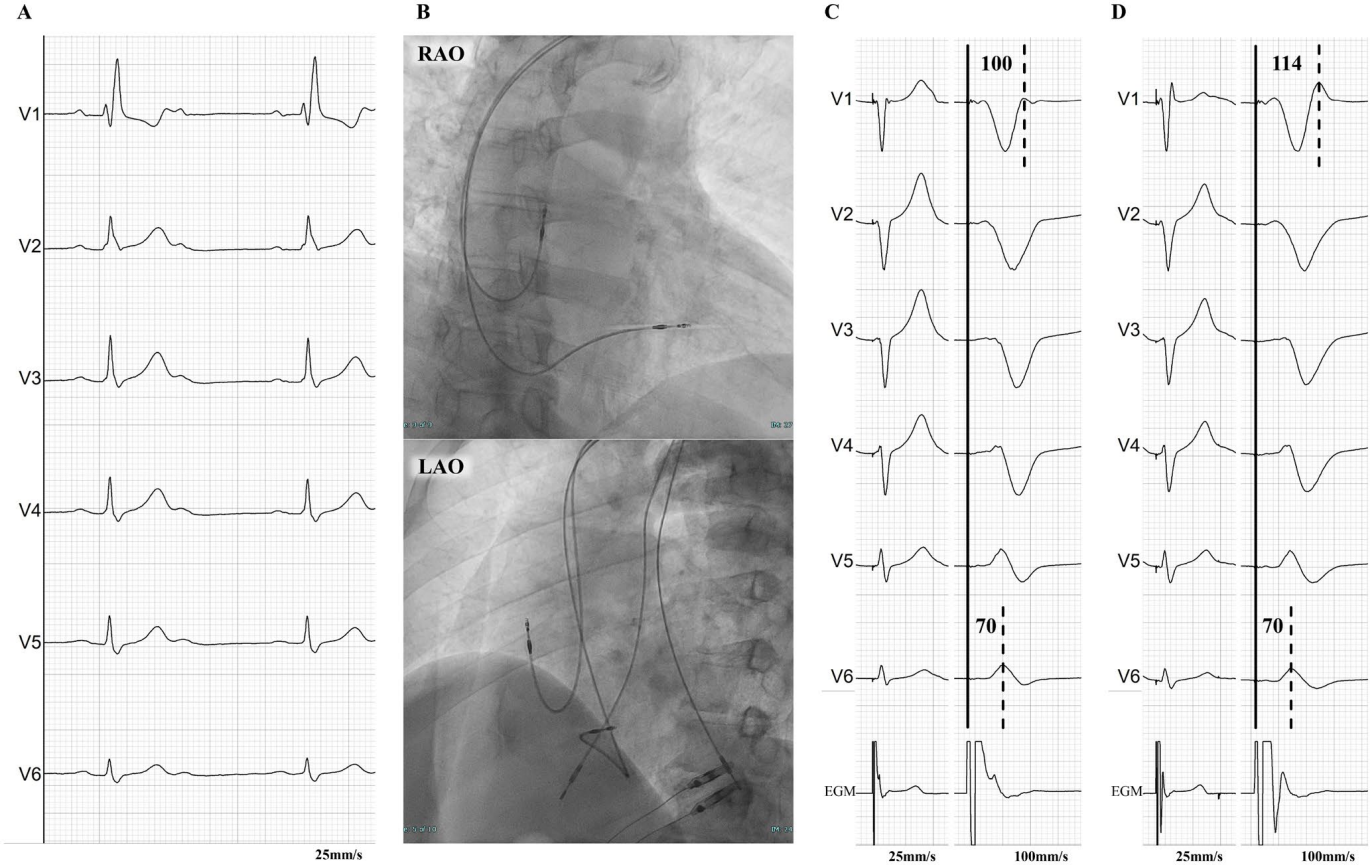
Chest leads of a 12‐lead electrocardiogram during implantation (A, C, D) and fluoroscopic image (B) in Case 1. (A) Intrinsic rhythm with 2:1 AVB and RBBB. QRSd was 178 ms. (B) Fluoroscopic RAO 30° view (top) and LAO 60° view (bottom) during implantation. (C) NS‐LBBP. Stim‐V6RWPT was 70 ms and Stim‐V1RWPT was 100 ms. (D) S‐LBBP. Stim‐V6RWPT was 70 ms, Stim‐V1RWPT was 114 ms, and a discrete potential after the pacing stimulus was observed in the endocardial EGM. AVB, atrioventricular block; EGM, electrogram; LAO, left anterior oblique; NS‐LBBP, nonselective left bundle branch pacing; RAO, right anterior oblique; RBBB, right bundle branch block; S‐LBBP, selective left bundle branch pacing; Stim‐V1RWPT, interval between stimulus artifact and peak of the R wave in lead V1; Stim‐V6RWPT, interval between stimulus artifact and peak of the R wave in lead V6; QRSd, QRS duration.

Case 2 was a 77‐year‐old female patient who underwent pacemaker implantation for a diagnosis of tachycardia‐bradycardia syndrome and paroxysmal atrial fibrillation. A preoperative 12‐lead electrocardiogram showed no conduction disturbance and a narrow QRS morphology (Figure 2A). We implanted a SelectSecure 3830 lead (Medtronic, Minneapolis, MN) using a C315 HIS catheter (Medtronic) and attempted LBBAP (Figure 2B); however, RBBB developed during lead implantation (Figure 2C). Although a central block (i.e., an intra‐Hisian block) cannot be excluded as the cause of the RBBB, the block occurred when the delivery catheter was positioned against the RV septum, suggesting a peripheral RBBB caused by mechanical injury to the RBB (i.e., an infra‐Hisian block) [2]. During the threshold test with bipolar pacing, three consecutive QRS morphologies were observed, although all showed a QRR′ pattern (Figure 2D–F). Even with progressively lower pacing outputs, the Stim‐V6RWPT remained unchanged at approximately 70 ms. The Stim‐V1RWPT was prolonged from 89 to 110 ms, whereas the interval from the pacing stimulus to the peak of the R′ wave in lead V1 (Stim‐V1R'WPT) remained unchanged at 132 ms; with a further reduction in pacing output, the Stim‐V1RWPT remained unchanged at 110 ms, and the Stim‐V1R'WPT was prolonged from 132 to 144 ms (Figure 2D–F). Because endocardial electrogram data were not available, a LBB potential during intrinsic conduction and a discrete potential after the pacing stimulus during S‐LBBP could not be confirmed. However, based on the changes in QRS morphology observed during the threshold test, the findings suggested NS‐LBBP with anodal capture (Figure 2D), NS‐LBBP (Figure 2E), and S‐LBBP (Figure 2F), respectively. A 12‐lead electrocardiogram on the day after implantation showed the same narrow QRS complex as before implantation, suggesting the recovery of RBB conduction. The QRS morphology during LBBAP showed a QR pattern, and the R′ wave observed at implantation was no longer present (Figure 2G). During the threshold test with bipolar pacing, the transition from NS‐LBBP to S‐LBBP was not observed.

**FIGURE 2:**
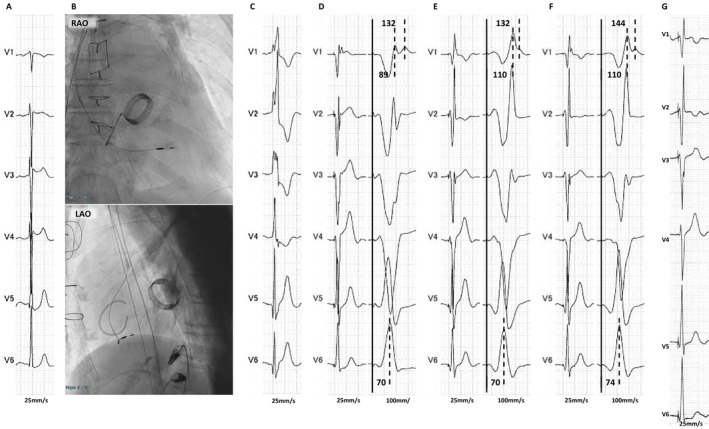
Chest leads of a 12‐lead electrocardiogram during implantation (A, C–F), fluoroscopic image (B) and chest leads of a 12‐lead electrocardiogram on the day after implantation (G) in Case 2. (A) Intrinsic rhythm with no conduction disturbance and a narrow QRS morphology. QRSd was 80 ms. (B) Fluoroscopic RAO 30° view (top) and LAO 60° view (bottom) during implantation. (C) Presumed peripheral RBBB associated with mechanical injury to the RBB related to the delivery catheter. QRSd was 140 ms. (D) NS‐LBBP with anodal capture in the presence of peripheral RBBB. Stim‐V6RWPT was 70 ms, Stim‐V1RWPT was 89 ms and Stim‐V1R'WPT was 132 ms. (E) NS‐LBBP in the presence of peripheral RBBB. Stim‐V6RWPT was 70 ms, Stim‐V1RWPT was 110 ms and Stim‐V1R'WPT was 132 ms. (F) S‐LBBP in the presence of peripheral RBBB. Stim‐V6RWPT was 70 ms, Stim‐V1RWPT was 110 ms and Stim‐V1R'WPT was 144 ms. (G) LBBAP after recovery of RBB conduction. LBBAP, left bundle branch area pacing; RBB, right bundle branch; Stim‐V1R'WPT, interval between stimulus artifact and peak of the R′ wave in lead V1. Other abbreviations as in Figure 1.

In Case 1, RBBB was corrected by LBBAP. In Case 2, the paced QRS morphology during LBBAP changed from a QRR′ pattern to a QR pattern due to the improvement of RBB conduction. Therefore, our findings suggest the possibility that electrical activation during LBBAP may penetrate into the RBB. The most straightforward mechanism by which the RBB is activated during LBBAP is the direct anodal capture of the RBB by the ring electrode. In addition, a previous study has suggested the existence of transverse interconnections between the RBB and the LBB [3], and it is possible that electrical activation penetrates into the RBB through such interconnections. Both the LBB and RBB arborize deeply within the interventricular septum (i.e., the septal branches of the LBB and RBB), and therefore may be captured during LV septal pacing [4]. Therefore, five possible mechanisms can be considered by which electrical activation penetrates into the RBB during LBBAP:

1. Direct anodal capture of the RBB by the ring electrode (Figure 3A).
2. Conduction from the LBB to the RBB through a transverse interconnection between them (Figure 3B).
3. Conduction from the septal branches of the LBB to the septal myocardium, with subsequent penetration into the RBB via its septal branches (Figure 3C).
4. Penetration from the LV septal myocardium into the RBB via its septal branches (Figure 3D).
5. Penetration from the LV septal myocardium into the LBB via its septal branches and subsequent conduction to the RBB through a transverse interconnection between the LBB and RBB (Figure 3E).

**FIGURE 3:**
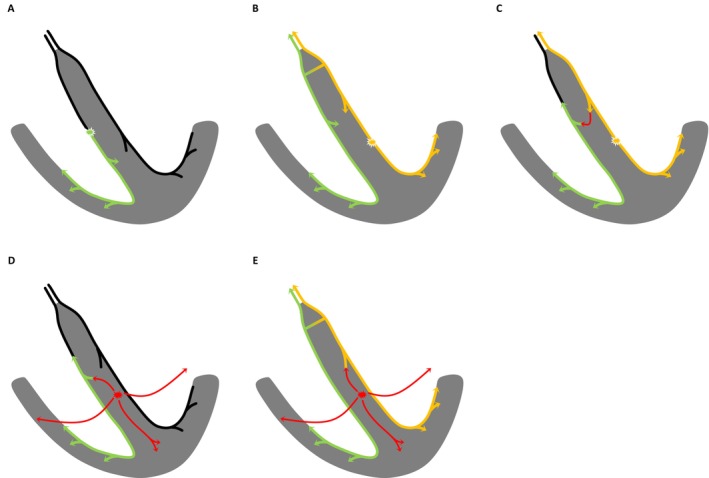
A schematic illustrating the mechanism of electrical activation penetrating the RBB in LBBAP. The yellow arrows indicate LBB conduction, the green arrows indicate RBB conduction, and the red arrows indicate myocardial conduction. (A) Direct anodal capture of the RBB by the ring electrode. (B) Conduction from the LBB to the RBB through a transverse interconnection between them. (C) Conduction from the septal branches of the LBB to the septal myocardium, with subsequent penetration into the RBB via its septal branches. (D) Penetration from the LV septal myocardium into the RBB via its septal branches. (E) Penetration from the LV septal myocardium into the LBB via its septal branches and subsequent conduction to the RBB through a transverse interconnection between the LBB and RBB. LBB, left bundle branch; LV, left ventricular. Other abbreviations as in Figures 1 and 2.

It should be noted that the two cases presented in this study were analyzed under the assumption of LBBP. Accordingly, the mechanisms illustrated in Figure 3D,E, which involve LV septal pacing, are presented only as conceptual illustrations to facilitate understanding of potential septal conduction pathways and should not be interpreted as mechanisms definitively demonstrated in the present cases.

Although an accurate assessment would ideally require verification of the propagation pattern using a mapping catheter, the presumed activation sequences in Cases 1 and 2 are illustrated in Figure 4 (Case 1: Figure 4A–C; Case 2: Figure 4D–J) based on the above considerations.

**FIGURE 4:**
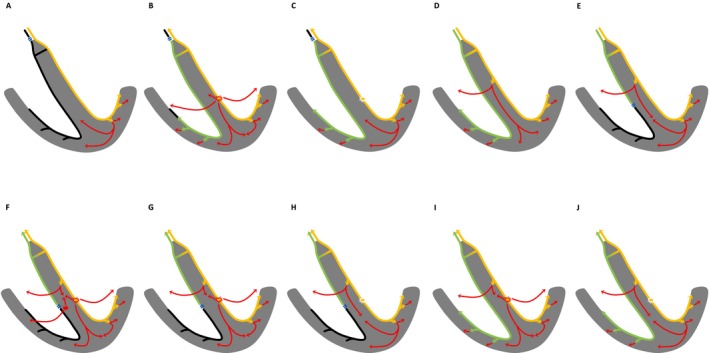
The estimated pattern of electrical activation in Case 1 (A–C) and Case 2 (D–J). The yellow arrows indicate LBB conduction, the green arrows indicate RBB conduction, and the red arrows indicate myocardial conduction. (A) Intrinsic rhythm with central RBBB. (B) NS‐LBBP in the presence of central RBBB. (C) S‐LBBP in the presence of central RBBB. (D) Intrinsic rhythm with no conduction disturbance and a narrow QRS morphology. (E) Presumed peripheral RBBB associated with mechanical injury to the RBB related to the delivery catheter. (F) NS‐LBBP with anodal capture in the presence of peripheral RBBB. (G) NS‐LBBP in the presence of peripheral RBBB. (H) S‐LBBP in the presence of peripheral RBBB. (I) NS‐LBBP after recovery of RBB conduction. (J) S‐LBBP after recovery of RBB conduction. Abbreviations as in Figures 1 and 2.

In Case 1, RBBB was corrected with both NS‐LBBP (Figure 1C) and S‐LBBP (Figure 1D). We speculated that the RBBB in this case was of the central type, such as a conduction disturbance within the His bundle (Figure 4A) [2], and the electrical activation of LBBP penetrated into the RBB distal to the block site, possibly through a unidirectional transverse interconnection from the LBB to the RBB (Figure 4B,C). Furthermore, as the pacing changed from NS‐LBBP (Figures 1C and 4B) to S‐LBBP (Figures 1D and 4C), the Stim‐V1RWPT was prolonged, suggesting that activation through the LV septal myocardium propagates to the RV earlier than activation through the RBB.

In Case 2, the RBBB developed when the delivery catheter was positioned against the RV septum; therefore, although a central RBBB cannot be excluded, the findings are consistent with the possibility of a peripheral RBBB related to mechanical injury to the RBB (Figures 2C and 4E) [2]. When the peripheral RBB was blocked, the paced QRS morphology in lead V1 showed a QRR′ pattern (Figure 2D–F), suggesting that delayed RV activation due to peripheral RBBB contributed to the appearance of the R′ wave. However, after recovery of RBB conduction, the R′ wave disappeared and the paced QRS morphology in lead V1 changed to a QR pattern (Figure 2G). Taken together, these findings suggest that electrical activation during LBBP may conduct to the RBB from the proximal side of the block, potentially via mechanisms such as a transverse interconnection from the LBB to the RBB (Figure 4F–H). Furthermore, as the pacing changed from NS‐LBBP (Figure 2E) to S‐LBBP (Figure 2F) in the presence of peripheral RBBB, the Stim‐V1RWPT remained unchanged, whereas the Stim‐V1R'WPT was prolonged. These findings suggest that the R wave in lead V1 during NS‐LBBP and S‐LBBP represents RV activation through the LBB (e.g., via septal branches of the LBB), that the R′ wave in lead V1 during NS‐LBBP represents RV activation through the LV septal myocardium, and that the R′ wave in lead V1 during S‐LBBP represents RV activation through the distal LBB (Figure 4G,H). In the intrinsic rhythm after the development of RBBB (Figure 2C) and during S‐LBBAP (Figure 2F), the initial Q wave in lead V6 was deeper than that observed during the intrinsic rhythm before the development of RBBB (Figure 2A). Although the presence of septal branches of the LBB cannot be directly confirmed, this finding may suggest their presence. When RBB conduction recovered, no change in the QRS morphology from NS‐LBBP to S‐LBBP was observed. This finding may indicate that selective LBB capture was lost, possibly due to lead micro‐dislodgement or changes in capture threshold on the day after implantation. On the other hand, it may also suggest that the R wave in lead V1 during NS‐LBBP and S‐LBBP represents a fusion of RV activation through the LBB (e.g., via septal branches of the LBB) and through the RBB, and that activation through the LV septal myocardium propagates to the RV later than these activations (Figure 4I,J). It should be noted that the above interpretation is based on the assumption that a transition from NS‐LBBP to S‐LBBP occurred in both Cases 1 and 2; therefore, this assumption itself may represent a limitation of the present study.

Similar to our Case 2, it has been reported that RBBB occurred at the infra‐Hisian level due to mechanical injury during LBBAP, and that the QRS morphology of LBBAP changed with the spontaneous recovery of RBB conduction [5]. This report suggested the possibility that the RV was activated via RBB conduction, raising potential concerns regarding the reliability of LBB capture indicators such as the V6‐V1 interpeak interval and V1RWPT [1], which assume that RV activation occurs through septal conduction. Based on the findings from the two cases we experienced, it is possible that these indicators may not be applicable for assessing LBB capture, depending on whether RV activation occurs via RBB conduction or LBB septal branch conduction, and on the relative timing of these activations compared with RV activation through the LV septum.

In conclusion, our findings suggest the possibility that electrical activation during LBBAP may penetrate into the RBB and contribute to RV activation. Accordingly, the validity of conventional indicators used to assess LBB capture during LBBAP may need to be reconsidered.

## Funding

The authors have nothing to report.

## Ethics Statement

The authors have nothing to report.

## Consent

Written informed consent was obtained from each patient.

## Conflicts of Interest

The authors declare no conflicts of interest.

## Data Availability

The data that support the findings of this study are available from the corresponding author upon reasonable request.
